# Insights into the use of mesenchymal stem cells in COVID-19 mediated acute respiratory failure

**DOI:** 10.1038/s41536-020-00105-z

**Published:** 2020-10-26

**Authors:** Nisha Durand, Jorge Mallea, Abba C. Zubair

**Affiliations:** 1 Laboratory Medicine and Pathology and Center for Regenerative Medicine, Mayo Clinic, Jacksonville, FL 32224 USA; 2 Department of Medicine, Division of Allergy, Pulmonary and Sleep Medicine, Mayo Clinic, Jacksonville, FL 32224 USA

**Keywords:** Mesenchymal stem cells, Mesenchymal stem cells

## Abstract

The emergence of severe acute respiratory syndrome corona virus 2 (SARS-CoV-2) at the end of 2019 in Hubei province China, is now the cause of a global pandemic present in over 150 countries. COVID-19 is a respiratory illness with most subjects presenting with fever, cough and shortness of breath. In a subset of patients, COVID-19 progresses to hypoxic respiratory failure and acute respiratory distress syndrome (ARDS), both of which are mediated by widespread inflammation and a dysregulated immune response. Mesenchymal stem cells (MSCs), multipotent stromal cells that mediate immunomodulation and regeneration, could be of potential benefit to a subset of COVID-19 subjects with acute respiratory failure. In this review, we discuss key features of the current COVID-19 outbreak, and the rationale for MSC-based therapy in this setting, as well as the limitations associated with this therapeutic approach.

## Introduction

The severe acute respiratory syndrome (SARS) corona virus 2 (SARS-CoV-2) disease (COVID-19) emerged in December 2019 in Wuhan, Hubei Province, People’s Republic of China when a cohort of patients with pneumonia of unknown etiology presented to local hospitals^1^. Since the initial outbreak of COVID-19 in Wuhan, the disease has since spread to over 150 countries with migratory epicenters that include Iran, Italy, Spain, and New York City, USA^2^. Given the widespread incidence of COVID-19, the World Health Organization (WHO) declared the current outbreak as a Public Health Emergency of International Concern (PHEIC) on January 30th 2020, and subsequently elevated the outbreak to pandemic status on March 11th 2020^3^. As of July 1, 2020, globally, there are about 10 million confirmed cases of COVID-19 and just over 500,000 fatalities have been reported^4^.

The causative agent of COVID-19, SARS-CoV-2, is an enveloped, positive-sense RNA coronavirus. Coronaviruses primarily cause respiratory and intestinal infections, and infect several species from bats to humans. Coronaviruses can have serious implications for public health: in addition to the current COVID-19 pandemic, they have also been responsible for SARS and Middle East respiratory syndrome, significant epidemics in the last two decades^5^. For the current outbreak of COVID-19, the initial patients who presented with the illness had all visited a local fish and wild animal market in Wuhan, Hubei Province, China^1^ which is indicative of a possible zoonotic transmission, but the exact mechanism which resulted in the first-in-person infection remains elusive. Propagation of the virus among humans is thought to occur primarily through three main transmission mechanisms: (I) contact transmission, (II) droplet transmission and (III) aerosol transmission^6^. The full genome sequence of SARS-CoV-2 has been elucidated, and it shares 79.6% sequence identity with SARS-CoV, and at the whole-genome level is 96% identical to a bat coronavirus. Entry into and subsequent infection of human alveolar epithelial cells by SARS-CoV-2 is mediated by the angiotensin-converting enzyme II (ACE2) receptor^7^.

For most patients with COVID-19 infections, initial symptoms include, but are not limited to fever, shortness of breath, cough and general malaise^8,9^. In the majority of patients, COVID-19 manifests as a mild-to-moderate disease and with conservative medical management, full recovery from the ailment is expected^10,11^. However, in a subset of high-risk patients; the elderly, and those with underlying medical conditions such as hypertension and diabetes, the disease can become critical, and is characterized by severe pneumonia, acute respiratory distress syndrome (ARDS), and multiple organ dysfunction^9,12–14^. Widespread inflammation, cytokine storm syndrome and a dysregulated immune response mediates the severe clinical manifestations seen in high-risk individuals with COVID-19^15–17^. In this cohort, disease- associated mortality is especially significant^9,10^.

There is no known cure for COVID-19, but given the prevalence of the disease, and the associated morbidity and mortality, various therapeutic modalities that might be effective against COVID-19 have been evaluated in clinical settings. Therapeutic agents assessed in COVID-19 patients thus far include, convalescent plasma^18^, antimalarials like Hydroxychloroquine^19^ and antivirals^20^. Consideration has also been given to the use of immunoregulatory agents in subjects with severe COVID-19^21–23^, since dysregulated and hyper-activated inflammatory responses tends to be a main driver of COVID-19-induced mortality^10^.

One such immunoregulatory agent with potential for COVID-19 subjects with critical disease is the mesenchymal stem cell (MSC). MSCs are multipotent stromal cells that induce immunomodulation and regeneration, and undergo tri-lineage differentiation when stimulated in vitro^24,25^. Beyond their immunomodulatory and regenerative capacity, MSCs also exhibit antimicrobial activity^26^ and an ‘immune privileged’ phenotype, which facilitates allogeneic transplantation in the absence of rejection^27^. MSCs have been isolated from multiple tissue types including bone marrow, adipose tissue and umbilical cord, and their functional effects are primarily mediated by paracrine signaling via secretion of cytokines and chemokines^28–30^.

In clinical settings, while the safety profile of MSCs has been well-established, often times, the efficacy data obtained in pre-clinical models have not been recapitulated in most cases when MSCs have been administered to human cohorts. MSC therapeutic effectiveness is likely limited to a niche of immunological disorders and immune-mediated illnesses such as graft-versus-host-disease, for which MSCs have demonstrated varying levels of efficacy in Phase 1/2 clinical studies^31,32^. In multiple pre-clinical studies, MSCs have been shown to be effective against ARDS and acute lung injury (ALI)^33,34^, both of which are severe clinical manifestations of COVID-19, and while, administration of MSCs to subjects with ARDS was well tolerated^35,36^, efficacy data at the clinical level is not as compelling. To date, there are about 40 clinical trials registered on *clinicaltrials.gov* for evaluating the use of MSCs in subjects with COVID-19 (Table 1)^37^.

**Table 1:** Phase and cell source for MSC therapy for COVID-19 related conditions (Clinicaltrials.gov).

Clinical trial phase	Total number (%)	MSC source per phase					
		Umbilical cord/Wharton’s jelly	Bone marrow	Adipose tissue	Dental pulp	Olfactory mucosa	Unknown
1	8 (20.5)	2	3	1	1	0	1
1/2	12 (30.8)	8	1	1	1	1	0
2	16 (41.0)	7	2	5	0	0	2
2/3	1 (2.6)	0	0	0	0	0	1
3	1 (2.6)	0	1	0	0	0	0
Unknown	1 (2.6)	1	0	0	0	0	0
**Total # of MSC source (%)**		**18 (46.2)**	**7 (17.9)**	**7 (17.9)**	**2 (5.1)**	**1 (2.6)**	**4 (10.3)**

In this review, we critically examine the mechanisms that render MSCs potentially beneficial for a subset of COVID-19 subjects with acute respiratory failure; the limitations associated with this therapeutic modality and the current status of COVID-19-relavant MSC clinical studies.

## COVID-19

### Pathogenesis of COVID-19 mediated acute respiratory failure and its clinical manifestation

COVID-19 subjects usually present with fever, cough, fatigue and myalgias. Some patients develop gastrointestinal symptoms like diarrhea and nausea. Shortness of breath is present in 80% of the patients with COVID-19 that require hospitalization^38^. For most subjects with a non-severe manifestation of the disease, a full recovery is expected. However, a subset of patients with COVID-19 develop serious complications in the course of their disease. These complications commonly require admission to the intensive care unit (ICU), and include ARDS, cardiomyopathy, acute kidney injury, acute liver injury and shock^17,39,40^. It is estimated that 5% of patients with COVID-19 will need admission to the ICU, with the majority of them requiring mechanical ventilation^41^. ARDS associated with COVID-19 usually appears 1–2 weeks after the onset of symptoms, and is associated with a higher mortality. The mortality from COVID-19 is currently estimated at about 5%^4^.

### Immune response to COVID-19 infection

A well-orchestrated innate immune response is the initial defense mechanism against viral pathogens, but under some circumstances hyper-inflammatory responses can occur during viral infections^5^. In line with this notion of hyper-inflammatory immune responses, the most dire complications that arise from COVID-19 such as ARDS and acute cardiac injury are associated with widespread inflammation. In a study of 1099 COVID-19 patients, most patients had elevated levels of C-reactive protein (CRP), an inflammatory biomarker^39^. When compared to patients who had recovered from COVID-19, those who succumbed to the disease had much higher levels of CRP in their blood^9^. Key inflammatory chemokines and cytokines such as interleukin (IL)-6, IL-8, tumor necrosis factor alpha (TNF-α), monocyte chemoattractant protein-1 (MCP-1) and macrophage inflammatory protein 1 alpha (MIP-1α) are elevated in COVID-19 subjects, and this is typically more pronounced in patients with severe manifestations of the disease^9,15,17,42^. In addition to the changes in the levels of inflammatory markers, alterations in immune cell quantity were also observed in COVID-19 patients. Lymphocyte counts are decreased in COVID-19 subjects and this is more pronounced in the subset of patients with severe disease^15,39^. On the other hand, in critically ill COVID-19 patients, elevated neutrophil counts have been reported^15,17^. Increased quantities of pro-inflammatory chemokines and cytokines, in conjunction with accompanying shifts in immune cell numbers is indicative of a dysregulated immune response in COVID-19 subjects, especially for those with critical illness. This dysregulated and hyper-activated immune response has been an area of focus for targeting COVID-19.

### Therapeutic interventions

Treatments used to combat COVID-19 thus far include intravenous (IV) antibiotics, systemic glucocorticoids, IV immunoglobulins, convalescent plasma and antiviral therapy^9,39,43^. The antimalarial drug chloroquine has also received much attention in the fight against COVID-19 and has already been evaluated in clinical trials^19^. Both chloroquine and the antiviral drug Remdesivir are effective in controlling SARS-CoV-2 infection in vitro^44^. However, the utility of agents like hydroxychloroquine for COVID-19 remains inconclusive as there have been reports of viral clearance associated with use of the drug^19^ but also data from multiple reports which indicate otherwise^45,46^. While drugs like chloroquine^44^ and immunoglobulins^47^ target the viral life cycle, a subset of drugs (glucocorticoids) used in COVID-19 subjects target inflammation. This is particularly important since ARDS, the most life-threatening complication associated with COVID-19, is primarily an inflammatory based condition. Targeting this aspect of the disease is crucial, and therapeutic agents like MSCs, which can counteract aggravated immune responses, should be considered in efforts geared towards the mitigation of COVID-19, particularly in those subjects with critical disease. Beyond immunomodulation, the regenerative potential and antimicrobial properties of MSCs would also be of practical importance in the setting of COVID-19.

## Rationale for MSC therapy to manage COVID-19 mediated acute respiratory failure: evidence from pre-clinical studies

### Immunomodulation

**Fig. 1:**
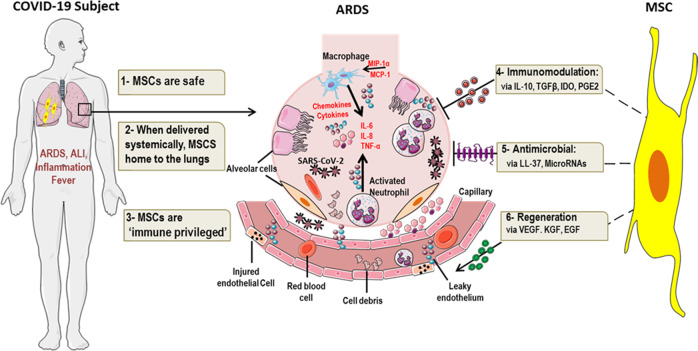
Rationale for MSC therapy in COVID-19 subjects with critical illness. Subjects with critical COVID-19 can develop serious inflammatory-mediated complications like ARDS. Immune cells like macrophages and neutrophils are activated, pro- inflammatory cytokines (IL-6, TNF-α) are released, and endothelial cells are injured in the lungs of these subjects. In this setting, MSCs could be of potential benefit since they are (I) Safe, (II) home to the lungs, (III) ‘immune privileged’, (IV) immunomodulatory, (V) antimicrobial and (VI) regenerative. Created using elements of Servier Medical Art by Servier, licensed under CC BY 3.0 (https://smart.servier.com/).

Seminal studies by Meisel et al.^48^ defined an anti-inflammatory role for MSCs when they showed that MSCs derived from bone marrow could inhibit T cell responses in a manner which was dependent on indoleamine 2, 3-dioxygenase. Since then, countless other studies have demonstrated the anti-inflammatory properties of MSCs. MSCs secrete anti-inflammatory chemokines and cytokines such as IL-10, transforming growth factor beta and Prostaglandin E2 (PGE2) (Fig. 1)^49–51^. Transcriptionally, MSCs regulate inflammation via *Heme Oxygenase-1 (HO-1)*^52^ and *Inducible T Cell Costimulator Ligand* (*ICOSL)*^53^. In COVID-19-relevant animal models, administration of MSCs have resulted in amelioration of disease symptoms. In liposaccharide (LPS)-induced ARDS in mice, treatment with MSCs resulted in attenuation of neutrophil infiltration, collagen deposition and fibrosis^54^. In a similar model, administration of MSCs protected mice from LPS-induced ALI by inhibiting macrophages in a manner that was dependent on the secretion of PGE2, granulocyte-macrophage colony-stimulating factor (GM-CSF) and IL-13^51^. In addition to secreting anti-inflammatory molecules themselves, MSCs can also antagonize the release of certain pro-inflammatory cytokines. This was demonstrated by Xu et al.; in a rodent model of oleic acid-induced ALI, IV transplantation of MSCs reduced the concentration of pro-inflammatory TNF-α in blood plasma and lung tissue extracts. Conversely, a concomitant increase in the level of anti-inflammatory IL-10 in these animals following MSC treatment was observed^55^. MSCs also govern the specific types of immune cells that are recruited to sites of injury; biased towards the infiltration of tolerogenic cell types like regulatory T cells. In a recent study by Wang et al.^56^, an improvement in LPS-induced ALI mediated by MSCs was dependent on the regulation of the balance between regulatory T cells and Th17 cells. Increased regulatory T cells were also observed in a mouse model of lung contusion following IV MSC administration^57^. The effectiveness of MSCs in mediating anti-inflammatory responses in the pulmonary space has also been recapitulated in large animal models. The severity of ALI as shown by improved oxygenation, was reduced in a sheep model of bacterial pneumonia following IV delivery of MSCs^58^.

COVID-19 infection has been shown to trigger a pro-inflammatory state. Huang et al. reported that initial plasma analysis of 41 patients infected with the novel coronavirus in Wuhan, China, shows that IL-1 beta, IL-1 receptor antagonist, IL-7, IL-8, IL-9, IL-10, basic fibroblast growth factor (FGF), granulocyte-colony stimulating factor (G-CSF), GM-CSF, interferon gamma (IFN-γ), interferon-inducible protein 10 (IP10), MCP-1, MIP-1α, MIP-1β, platelet-derived growth factor (PDGF), TNF-α, and vascular endothelial growth factor (VEGF) were elevated in hospitalized patients when compared with healthy controls. Levels of IL-2, IL-7, IL-10, G-CSF, IP10, MCP-1, MIP-1α, and TNF-α were higher in patients requiring ICU care^59^. Ruan et al. reported higher levels of CRP and IL-6 in the patients that died with COVID-19 when compared to the patients that were discharged from the hospital. They concluded that the COVID-19 associated mortality may likely be due to a virus-activated “cytokine storm syndrome”^60^.

Though it is well-established that MSCs attenuate inflammatory responses, we and others have shown that MSCs secrete various inflammatory cytokines, particularly IL-6. We have actually shown that IL-6 plays a critical role in MSC functioning^61^. IL-6 function is dependent on how its signal is transmitted to target cells. IL-6 acts via its classical signaling pathway by binding to its membrane bound receptor (IL-6R), which then dimerizes with glycoprotein 130 (gp130). Activation of the classical IL-6 signaling was shown to induce anti-inflammation and epithelial cell regeneration. In addition, IL-6 can act via its soluble receptor (sIL-6R) coupled with gp130 protein to act on non-IL-6R expressing target cells. This is referred to as the trans-signaling pathway. It has been suggested that IL-6 trans-signaling is pro-inflammatory^62^.

### Regeneration and repair

While the immunomodulatory effects of MSCs are critical for counteracting the aggravated immune response, repair to damage and injured cells and tissues would also be required in the setting of ARDS. MSCs secrete multiple factors implicated in regeneration and modulation of tissue injury (Fig. 1); epidermal growth factor (EGF), PDGF, FGF, hepatocyte growth factor (HGF), VEGF and insulin-like growth factor^29,63–65^. Inhibition of key regenerative mediators in MSCs abrogated their ability to attenuate lung injury in vivo, as demonstrated by Yang et al. who used a sophisticated series of experiments to make this determination. They showed that while MSC transplantation in mice reduced lung permeability, protected lung epithelium from apoptosis, facilitated vascular endothelium (VE)–cadherin recovery and reduced lung injury, these beneficial effects were significantly diminished when VEGF expression was inhibited in the transplanted MSCs^66^. MSCs can also induce the expression of regenerative mediators in the setting of ALI. In a similar study, reduced HGF expression in MSCs impaired their ability to protect against lung fibrosis in vivo, in a mouse model of Bleomycin-induced pulmonary fibrosis. Furthermore, it was shown that inhibition of pulmonary cell apoptosis mediated by MSC conditioned medium (CM) was HGF dependent^67^. In addition to secreting these regenerative molecules themselves, MSCs can also induce the expression of these factors in specific cells and tissues. IV administration of MSCs in a mouse model of LPS-induced ALI resulted in upregulated expression of keratinocyte growth factor^68^, an important mediator of alveolar repair^69^. In a rodent model of radiation-induced pulmonary fibrosis, administration of MSCs preserved the architecture of irradiated lungs, and this was partly mediated by endogenous expression of HGF^70^. Repair of the pulmonary blood-air barrier in a rodent model of phosgene-induced lung injury has also been shown to be mediated by MSCs^71^.

### Antimicrobial properties

There is a growing body of evidence which implicates an antimicrobial role for MSCs (Fig. 1); a characteristic that would be applicable for combating COVID-19. Multiple of the studies which have evaluated the nature of MSC antimicrobial activity have revealed a role for antimicrobial peptides (AMPs) in the process. AMPs are a diverse class of low molecular weight proteins produced as a first line of defense against pathogens such as bacteria, fungi and viruses^72^. In revolutionary work by Krasnodembskaya et al. it was shown that the antibacterial effect of MSCs was partly mediated by LL-37, an AMP. Using a series of well-designed experiments, they showed that in vitro, MSCs as well as MSC-CM inhibited the growth of both Gram-negative and Gram-positive bacteria. In addition, in a mouse model of *E. coli-*induced pneumonia, administration of MSCs reduced bacterial growth in bronchoalveolar lavage (BAL) fluid and lung homogenate, both of which were attenuated when a neutralizing antibody to LL-37 was introduced^26^. In a similar study of *E. coli*-induced pneumonia, MSC therapy decreased lung bacterial burden and this was accompanied by elevated alveolar concentrations of LL-37^73^. MSC-induced bacterial clearance mediated by LL-37 has also been demonstrated in mouse models of cystic fibrosis^74^. In a rodent model of *E. coli*-induced ARDS, MSC administration was associated with reduced formation of *E. coli* colony forming units in BAL^75^. Beta-defensin 2 (BD2), is another AMP secreted by MSCs; in a mouse model of *E. coli*-induced ALI, MSC administration resulted in enhanced bacterial clearance in a manner consistent with augmented BD2 secretion^76^. In contrast to the multiple studies focusing on the antibacterial activity of MSCs, reports of specific antiviral activity as it relates to MSCs have been less forthcoming, nevertheless, MSC-derived exosomal microRNAs have been shown to inhibit Hepatitis C virus infection in vitro^77^. MSCs also inhibit inflammasome activation in the presence of Coxsackievirus B3^78^. Finally, while studies of influenza virus-induced ALI in mice have demonstrated that MSC administration reduced lung injury and pulmonary inflammation, and restored alveolar fluid clearance^79–81^, the actual ability of MSCs to inhibit viral replication in relevant animal models has not been demonstrated.

Paradoxically, while multiple studies have shown a definite antimicrobial role for MSCs, there are reports which indicate otherwise. In a study published by Schwartz et al., they demonstrated that MSC treatment in mice infected with *Mycobacterium bovis* promoted bacterial growth in the spleen. Suppression of bacterial growth was only achieved after the MSCs were preconditioned with TLR-3 ligand^82^. This data is telling, but not entirely unexpected, as MSCs are actively involved in immunomodulation. This however raises important questions relating to MSC function in inflammatory-mediated viral illnesses such as COVID-19; would the abatement of the cytokine storm syndrome be achieved at the cost of increased viral load? The answers to these critical questions remain unknown.

### First pass entrapment

As described above, MSCs exhibit several characteristics in vitro and in vivo, which if mirrored in human cohorts could be of potential therapeutic benefit for a subset of patients with severe manifestations of COVID-19. However, with all drug therapeutics, the mode of drug delivery and targeting strategy needs to be given serious consideration. Dependent on the indication, MSCs have been delivered via multiple routes of administration, from direct injection to IV delivery^83^. In the context of COVID-19-associated ARDS, MSCs need to exert their therapeutic effects in the lungs. In pioneering studies by Fisher et al.^84^ it was shown that when MSCs are delivered intravenously, the majority of cells remained trapped in the lungs, with limited quantities reaching other major organs like the heart, kidneys, and liver. This pulmonary first-pass effect has been investigated extensively; in a rodent model of silicosis fibrosis, when fluorescently labeled MSCs were delivered by IV, fluorescence intensity in the lungs peaked 6 h post injection; and 15 days post injection, fluorescence signals could still be detected in the lungs, albeit at much lower levels^85^. While this pulmonary entrapment of MSCs may be a barrier to certain types of MSC-based therapies, in the context of COVID-19-related ARDS, it is ideal (Fig. 1).

### Outcome of clinical studies using MSCs

Most of the data demonstrating the beneficial effects mediated by MSCs have been obtained from studies conducted in vitro and in vivo; however, in clinical settings the therapeutic efficacy of MSC-based therapy has not been readily demonstrated. Although multiple MSC-based clinical studies have been completed in the pulmonary space for conditions like chronic obstructive pulmonary disease (COPD), ARDS, emphysema and obstructive chronic lung allograft dysfunction^36,86–90^, these studies are primarily Phase 1 trials which evaluate safety and feasibility. Data from these early-stage clinical trials demonstrate that administration of MSCs was safe and well-tolerated with no serious adverse events being reported. Nonetheless, in some of these early stage-clinical studies, positive indicators of MSC therapy have been reported. In subjects with ARDS, when MSCs were administered, 5 days post infusion, serum levels of surfactant protein D, an ALI biomarker, were significantly lowered as compared to baseline levels pre-infusion. However, in this same study, no significant differences between the treatment and control groups in the PaO_2_/FiO_2_ ratio were observed^91^. Administration of MSCs to subjects with severe emphysema resulted in increased CD31 expression, and CD3^+^ and CD4^+^ T cells, but the significance of these changes in the context of emphysema pathogenesis remains elusive. More importantly, in this study, no macroscopic or molecular evidence for repair of emphysematous lesions were detected following MSC infusion^88^. In a comprehensive study of two patients with severe ARDS for which MSCs were administered on a compassionate basis, several beneficial outcomes were reported: respiratory, hemodynamic and multi-organ failure were resolved following MSC treatment. Moreover, there were reduced levels of systemic and pulmonary markers of inflammation including epithelial apoptosis, alveolar-capillary fluid leakage, and pro-inflammatory chemokines, microRNAs and cytokines^92^. While the aforementioned studies are primarily Phase 1 studies which were not necessarily designed to evaluate efficacy, the efficacy data from these studies is weak.

Convincing efficacy data from larger scale clinical studies for lung-related illnesses using MSCs are limited. In a randomized double-blinded study of 62 COPD subjects, systemic delivery of MSCs resulted in a significant decrease in levels of circulating CRP in patients who had elevated CRP levels at initiation of the study^90^. As noted above, COVID-19 subjects with critical illness have elevated CRP levels in comparison to their counterparts with a non-severe form of the disease^9,39^. While modulation of inflammatory markers in response to MSC treatment might be a beneficial outcome in the setting of systemic inflammation, MSC treatment did not alter important clinical parameters such as COPD exacerbations, pulmonary function tests (PFT) or quality of life indicators. In a larger scale, Phase 2, multi-centered clinical trial for patients with moderate to severe ARDS, while post-hoc analyses revealed a trend towards improvement in oxygenation index as well as a reduction in endothelial injury as determined by reduced plasma concentrations of angiopoietin-2, in the MSC-treatment group compared to the control group, more relevant clinical outcomes such as a ventilator-free days did not significantly vary between the two groups^35^. The positive indicators associated with MSC administration reported in the aforementioned studies are mostly related to auxiliary measurements such as modulation of specific biomarker levels, instead of objective clinical indicators of responsiveness such as mortality, PFT, days hospitalized or ventilator-free days.

In yet another larger scale, Phase 1/2 study in patients with Bronchiolitis obliterans syndrome, positive clinical outcomes in response to MSC treatment were reported. For this specific study, 81 patients were enrolled, among which 49 received MSCs in addition to their standard therapy while 32 did not. After 3 months of treatment, 71% of patients in the MSC group had achieved response (PFT improvement and steroid sparing) as compared to 44% in the non-MSC group (*p* = 0.013). Clinical improvement was also accompanied by an increase in IL-10 producing CD5^+^ B cells^93^. The varied clinical outcomes reported in the larger scale clinical studies suggest that the beneficial effects of MSCs may be limited to specific pulmonary ailments and not all variations of lung-mediated illnesses, and that results from these studies can be influenced by multiple variables such as MSC dosing and drug preparation regimens.

MSCs have already been delivered to subjects with COVID-19, and the number of MSC-based clinical trials in these subjects is on the rise. To date, there are almost 40 clinical trials (Tables 1 and 2), across multiple phases for evaluating MSC therapy in COVID-19 subjects listed on *clinicaltrials.gov*^37^. The majority of these trials have not been completed and as such results from these studies have not been published. According to one case report, a three-time IV administration of umbilical cord MSCs to a critically ill COVID-19 subject was well tolerated and resulted in reduced serum CRP, normalization of white blood cell counts, and alleviation of pneumonia^94^. In another clinical study of ten subjects with COVID-19 pneumonia, 7 were administered MSCs, while the remaining 3 served as a placebo control. MSC transplantation was safe with no reports of infusion-related reactions or delayed hypersensitivity reported. In terms of efficacy, for one critically ill patient with severe pneumonia, MSC administration led to a significant and robust decrease in plasma CRP and an increase in oxygen saturation. Compared to the placebo control, subjects in the MSC group experienced normalization of immune cell populations, reduced serum TNF-α and increased IL-10. Of note, the investigators also went on to show that the transplanted MSCs lack ACE2 expression indicating that they are free of COVID-19 infection^95^. According to data from a recently published study, administration of Wharton’s Jelly-derived MSCs to a single subject with severe COVID-19 pneumonia resulted in resolution of fever and shortness of breath within two days of MSC transplantation and significant reductions in ground-glass opacity and pneumonia infiltration after six days. In this subject, improved clinical function was associated with increased T cell numbers, and reduced inflammatory mediators such as CRP, IL-6 and TNF- α^96^. Collectively, the data (Table 3) from these initial reports suggest that administration of MSCs to subjects with severe manifestations of COVID-19 appears to be beneficial in the resolution of major disease symptoms.

**Table 2:** Delivery method and number of cell doses for MSC therapy for COVID-19 related conditions (Clinicaltrials.gov).

Delivery method	Total number (%)	Number of doses to be administered					
		1	2	3	4	5	Unknown
Intramuscular injection	1 (2.6)	1*	0	0	0	0	0
Intravenous	36 (92.3)	5	6	10	5	2	8
Unknown	2 (5.1)	1	1	0	0	0	0
**Total # of doses (%)**		**7 (17.9)**	**7 (17.9)**	**10 (25.6)**	**5 (12.8)**	**2 (5.1)**	**8 (20.5)**

1*—Subjects randomized to high dose cohort may receive an additional dose.

**Table 3:** Published clinical studies for MSC use in COVID-19 related conditions.

Study title	Source of MSCs	Trial phase (sample size)	Autologous or allogeneic	COVID-19 condition	Location	Dose delivery	Outcome measures	Results	Ref.
Clinical remission of a critically ill COVID-19 patient treated by human umbilical cord mesenchymal stem cells	Umbilical cord	N/A (1)	Allogeneic	Critically ill type COVID-19	China	3 doses of 5 × 10^7^ cells, IV administration	Tolerability, Clinical improvement	Well tolerated, reduced CRP, pneumonia alleviation	^94^
Transplantation of ACE2- Mesenchymal Stem Cells Improves the Outcome of Patients with COVID-19 Pneumonia	Unknown	N/A (10)	Allogeneic	Covid-19 pneumonia (common, severe, critically severe)	China	1 × 10^6^ cells/kg, IV administration	Adverse effects, clinical outcomes, change in inflammatory and immune function level	No adverse events, reduced CRP and TNF-α, increased IL-10, normalization of immune cell populations	^95^
Intravenous infusion of human umbilical cord Wharton’s jelly-derived mesenchymal stem cells as a potential treatment for patients with COVID-19 pneumonia	Wharton’s Jelly	N/A (1)	Allogeneic	Covid-19 pneumonia, critically severe	China	1 × 10^6^ cells/kg, IV administration	Safety, clinical improvement	No adverse events, resolution of fever and shortness of breath, decreased inflammatory factors (TNF-α and IL-6)	^96^

## Barriers to MSC therapy

As described above, MSCs exhibit multiple beneficial effects and data from countless studies conducted in vivo in relevant animal models have shown that MSCs are effective against conditions such as ARDS and *E. coli-*induced pneumonia. However, translation of these positive findings in pre-clinical studies has been difficult to replicate in clinical trials with human subjects, with many MSC trials failing to progress beyond Phase 1. There are many challenges associated with MSC therapy including low in vivo survival rates, dosing, cell isolation and growth strategies, and donor variability issues. (Refer to ref. ^97^ for a review focused on the challenges associated with MSC therapy).

As is relates to survivability, data obtained from pre-clinical studies indicate that when MSCs are administered in vivo, they do not survive long term^98^. Evaluation of MSC viability 24 h post transplantation in the infarcted myocardium of rats showed that almost 70% of the transplanted MSCs were dead^99^. Interestingly, the short half-life of MSCs does not seem to be directly related to their efficacy; Yang et al.^100^ showed that MSCs rescued hematopoiesis in mice despite rapid clearance after infusion. This apparent conundrum between MSC survivability and efficacy is one of the many unresolved issues in the MSC field. Similarly, while there are countless studies^28,29,63,66^ which demonstrate that MSC function is largely mediated by paracrine signaling, some reports have indicated otherwise. In addition to paracrine signaling, MSCs also rely on cell–cell contact and direct mitochondrial transfer to elicit their function^101^. This was shown by Rosado et al.^102^ who demonstrated that MSC immunosuppressive activity required for inhibition of B-cell proliferation was dependent on cell–cell contact between MSCs and T cells. This specific knowledge gap in MSC function and biology, likely contributes to the lack of effectiveness at the clinical level.

The use of inadequate animal models in a well-controlled setting which does not sufficiently mimic the natural course of human disease is another major contributing factor to the poor outcomes in MSC clinical trials. For example, many of the pre-clinical studies of pulmonary fibrosis in mice have relied on the use of bleomycin to induce an acute phase of the aliment^67^, however, for actual patients it is unlikely that their disease developed in such a manner. As such, there would be differences in the pathophysiology of the disease, and how it responds to treatment in these respective settings. While it is true that there are inherent limitations associated with the use of any animal model used for mimicking human disease, even with *perfect* animal models, there are other discrepancies which need to be addressed before successful translation of MSC therapy can be achieved.

The source of the MSCs, as well as the donor (allogeneic setting) from which they are derived can be a source of huge variability in MSC phenotype and function. We have previously shown that MSCs derived from different donors exhibit significant variation as it relates to cell growth and cytokine secretion profile^30^. In addition, MSCs derived from different tissue sources from the same donor also exhibit variability, as demonstrated by Wegmeyer et al.^103^ who showed that MSCs derived from umbilical cord and amniotic membrane from the same donor have different growth characteristics and morphology, and distinctive cytokine and growth factor secretion patterns. For clinical studies, selection of the ideal cell source and optimal donor for generation of the most potent cell product is particularly challenging. Beyond the variability among cell source and donors, variability in cell expansion protocols and cell preparation techniques, can also adversely affect the results of clinical studies. MSCs expanded in cell culture flatware have different growth kinetics compared to their counterparts expanded in automated 3D-bioreactor systems^104^. Clinical outcomes in MSCs trials are also influenced by cell preparation techniques. In the Phase 2 study for ARDS patients discussed above^35^, the investigators reported that there were substantial differences in MSC viability after preparation for infusion across the different sites participating in the trial. They also reported a significant viability-dependent effect on angiopoietin-2 concentrations in plasma 6 h post MSC administration.

Successful translation of MSC-based therapies in clinical settings continues to face significant challenges, with very few studies progressing to Phase 3. Though convincing data relating to MSC efficacy in larger cohort studies are limited, they do exist. According to data from a completed Phase 3 trial in which MSCs derived from Adipose Tissue were administered to subjects with Crohn’s disease, 50% of subjects in the MSC cohort achieved remission while only 34% in the control group did (*p* = 0·024)^105^. This study demonstrates that positive clinical outcomes in response to MSC treatment in large scale, Phase 3 studies are feasible, if thorough and detailed consideration is given to key parameters such as cell source and preparation techniques, in the design and execution of the study.

## Unique challenges of MSC therapy for COVID-19

We have discussed at length the beneficial effects of MSCs as it relates to their immunomodulatory, regenerative and antimicrobial nature, however, it is important to note that the majority of these findings were delineated in studies conducted in vitro, and in vivo models. As highlighted above, recapitulation of efficacy data obtained in animal models in human subjects remains a major barrier to successful translation of MSC therapy in clinical settings. This is even more relevant in the setting of COVID-19, as there is no pre-clinical efficacy data for the use of MSC in animal models of COVID-19 pneumonia. These pre-clinical studies are crucial, as they can provide valuable information on the consequences, both beneficial and deleterious of MSC administration specifically in the context of COVID-19 pneumonia. For example, while multiple studies have shown that MSC antimicrobial effects are mediated by LL-37, this molecule has also been associated with the pathogenesis of certain autoimmune disorders such as psoriatic arthritis^106^. The implication of this finding in the context of inflammatory mediated conditions like COVID-19 remains unknown.

Even in the absence of pre-clinical data specific to MSCs and COVID-19, MSCs have already been administered to COVID-19 subjects and there are multiple ongoing trials for use of MSCs in these patients (Tables 1 and 2). The data from the initial studies suggest that MSC administration to subjects with COVID-19 pneumonia is associated with alleviation of disease symptoms^94–96^. As depicted in Tables 1 and 2, for the ongoing and proposed clinical studies, there is considerable variation is terms of cell source and dosing regimen. Though administration of MSCs have already been associated with positive outcomes in COVID-19 subjects, best practices as it relates to formulation, cell source, dosing regimen and timing has not been established. These clinical studies (Tables 1 and 2) evaluating MSCs in COVID-19 subjects could resolve some of these key issues. Pre-clinical studies in relevant animal models of COVID-19 pneumonia could also assist in answering these questions.

## Summary and perspectives

COVID-19-associated mortality is increasing daily and as such there remains a critical and unmet need for effective therapeutic options for COVID-19 subjects with severe illness. In patients with critical manifestations of COVID-19, life-threatening complications such as ARDS and multiple organ dysfunction are mediated by extensive inflammation, cytokine storm syndrome, and dysregulated immune responses. MSCs because of their immunomodulatory, regenerative, and antimicrobial properties (Fig. 1) could render several therapeutic effects in the setting of COVID-19. While data from countless clinical studies have established that MSCs are safe, the data with regards to MSC efficacy hasn’t been as conclusive.

The safety of MSCs, the extensive pre-clinical efficacy data relating to their use in conditions with pathophysiologies analogous to that of COVID-19 pneumonia, and the initial reports of positive clinical indicators in response to MSC treatment in human cohorts with COVID-19, leads us to believe that MSCs are a potentially beneficial therapeutic option for COVID-19 subjects with critical manifestations of the disease which is governed by a hyper-inflammatory state. While we believe that MSC therapy may be potentially beneficial in this setting, we want to emphasize that MSCs cannot and should not be viewed as a panacea for COVID-19 and that administration of MSCs should not occur in the absence of grave disease complications such as hypoxic respiratory failure and ARDS, and only in the context of an approved clinical trial at no cost to the subjects.
